# How to engage stakeholders in research: design principles to support improvement

**DOI:** 10.1186/s12961-018-0337-6

**Published:** 2018-07-11

**Authors:** Annette Boaz, Stephen Hanney, Robert Borst, Alison O’Shea, Maarten Kok

**Affiliations:** 1 Faculty of Health, Social Care and Education, a partnership between Kingston University and St George’s, University of London, London, United Kingdom; 2 Health Economics Research Group, Brunel University London, Uxbridge, United Kingdom; 3 Erasmus School of Health Policy & Management, Rotterdam, The Netherlands; 4 VU University Amsterdam, Amsterdam, The Netherlands

## Abstract

**Background:**

Closing the gap between research production and research use is a key challenge for the health research system. Stakeholder engagement is being increasingly promoted across the board by health research funding organisations, and indeed by many researchers themselves, as an important pathway to achieving impact. This opinion piece draws on a study of stakeholder engagement in research and a systematic literature search conducted as part of the study.

**Main body:**

This paper provides a short conceptualisation of stakeholder engagement, followed by ‘design principles’ that we put forward based on a combination of existing literature and new empirical insights from our recently completed longitudinal study of stakeholder engagement. The design principles for stakeholder engagement are organised into three groups, namely organisational, values and practices. The organisational principles are to clarify the objectives of stakeholder engagement; embed stakeholder engagement in a framework or model of research use; identify the necessary resources for stakeholder engagement; put in place plans for organisational learning and rewarding of effective stakeholder engagement; and to recognise that some stakeholders have the potential to play a key role. The principles relating to values are to foster shared commitment to the values and objectives of stakeholder engagement in the project team; share understanding that stakeholder engagement is often about more than individuals; encourage individual stakeholders and their organisations to value engagement; recognise potential tension between productivity and inclusion; and to generate a shared commitment to sustained and continuous stakeholder engagement. Finally, in terms of practices, the principles suggest that it is important to plan stakeholder engagement activity as part of the research programme of work; build flexibility within the research process to accommodate engagement and the outcomes of engagement; consider how input from stakeholders can be gathered systematically to meet objectives; consider how input from stakeholders can be collated, analysed and used; and to recognise that identification and involvement of stakeholders is an iterative and ongoing process.

**Conclusion:**

It is anticipated that the principles will be useful in planning stakeholder engagement activity within research programmes and in monitoring and evaluating stakeholder engagement. A next step will be to address the remaining gap in the stakeholder engagement literature concerned with how we assess the impact of stakeholder engagement on research use.

**Electronic supplementary material:**

The online version of this article (10.1186/s12961-018-0337-6) contains supplementary material, which is available to authorized users.

## Background

Closing the gap between research production and research use is a key challenge for the health research system. Stakeholder engagement is being increasingly promoted across the board by health research funding organisations, and indeed by many researchers themselves, as an important pathway to achieving impact [1]. The literature is diverse, with a rapidly expanding but still relatively small number of papers specifically referring to ‘stakeholder engagement’, and a larger number of publications discussing issues that at least partly overlap with stakeholder engagement. Several of the papers explicitly analysing stakeholder engagement come from the field of environmental research (e.g. Jolibert and Wesselink [2], Phillipson et al. [3]). However, stakeholder engagement is also gaining traction in the health field. A recent supplement in this journal consolidated learning relating to tools and approaches to stakeholder engagement within the United Kingdom Department for International Development’s Future Health Systems research consortium [4]. In particular, in health, there is an important stream of analysis from North America. A review for the United States Agency for Healthcare Research and Quality drew on papers from a range of fields [5].

This opinion piece provides a short conceptualisation of stakeholder engagement, followed by ‘design principles’ that we put forward based on a combination of existing literature and new empirical insights from our recently completed longitudinal study of stakeholder engagement in research. We have drawn on a systematic literature search conducted to inform the wider study and in particular to conceptualise stakeholder engagement (Additional file 1).

### Literature review

#### Conceptualising stakeholder engagement: What does the literature say?

Stakeholders have been defined as “*individuals, organizations or communities that have a direct interest in the process and outcomes of a project, research or policy endeavor*” ([6], p. 5). In seeking to conceptualise stakeholders, Concannon et al. [7] developed the 7Ps Framework to identify stakeholders in Patient-Centered Outcomes Research and Comparative Effectiveness Research in the United States of America. The 7Ps are patients and the public, providers, purchasers, payers, public policy-makers and policy advocates working in the non-governmental sector, product makers, and principal investigators. The seven categories signal an overlap with the large literature on patient and public involvement (PPI) in research. However, our focus here is on multi-stakeholder engagement, where diverse groups of stakeholders take part in the research process. Deverka et al. [6] define engagement as “*an iterative process of actively soliciting the knowledge, experience, judgment and values of individuals selected to represent a broad range of direct interest in a particular issue, for the dual purposes of: creating a shared understanding; making relevant, transparent and effective decisions*” ([6], p. 5).

#### Roles, activities and phases of stakeholder engagement: What does the literature say?

There are additional issues about the definition of stakeholder engagement when the nature of the engagement activities is considered. For example, there are issues about how far co-creation/participatory action research approaches can be considered to be stakeholder engagement or something so far beyond the usual stakeholder engagement that they are really in a different category [8]. Similarly, there is a large and currently distinct literature on PPI in research [9], including the development of reporting guidelines such as GRIPP2 [10]. There are a number of parallels in the issues discussed in these literatures as well as some interesting differences (particularly in terms of power inequalities). However, herein, we conceptualise PPI as a subset of stakeholder engagement in-line with most of the literature, including Concannon et al. [7].

Most of the stakeholder engagement literature highlights the broad range of activities in which stakeholders can engage depending on their own skills and attributes and the capacity and wishes of the researchers conducting specific studies. At the broadest level of a research system, or research funding body, Lomas [11] claimed there were many activities in which stakeholders could be engaged in a ‘linkage and exchange’ approach for health services research. These were setting priorities, funding programmes, assessing applications, conducting research and communicating findings. The importance of engaging a wide range of stakeholders in priority-setting has often been emphasised. The pioneering study by Kogan and Henkel [12] analysed both the importance of engaging policy-makers in setting research agendas to meet their needs, and the obstacles to making the process work well. These obstacles included issues around how far the assessment of needs-based research should focus on the relevance and practical impact of the research as well as its scientific merit. Many of the more recent studies explicitly examining stakeholder engagement also set out a range of activities in which stakeholders may be involved. These are often related to phases of the research processes. Concannon et al. [7] provide a list of roles related to stages and used the identified roles in a subsequent review [13].

Knowledge translation (KT) is one of many terms used to describe efforts to ensure research evidence is used to inform decision-making [14]. Although the importance of engaging stakeholders in KT is recognised, it has been acknowledged that stakeholder engagement is often overlooked in favour of more conventional dissemination strategies [15]. Integrated KT has been developed as an approach to collaborative research in which researchers work with stakeholders who identify a problem and have the influence and sometimes authority to implement the knowledge generated through research [16, 17]. Grimshaw et al. [14] argue that different groups of stakeholders are likely to be engaged depending on the type of research that is being translated.

#### Assessing the impact of stakeholder engagement: What does the literature say?

A final consideration about the nature of the body of literature specifically on stakeholder engagement is that not only is it still quite limited in total, but there are also notable areas where authors claim it is particularly sparse. In particular, Hinchcliff et al. [18] examined the literature on multi-stakeholder health services research collaborations in an attempt to address the question of whether it was worth investing in them. They identified very few studies (Harvey et al’s. [19] 2011 evaluation of a Collaboration for Leadership in Applied Health Research and Care being one exception) and concluded that their generalisability was questionable. They therefore suggested that “*The lack of reliable evidence compels implementers to rely largely on trial and error, risking variable success*” ([18], p. 124).

The nature of engagement activity is less contentious than the arguments about its potential impact. Research impacts on non-academic audiences are defined by the United Kingdom Higher Education Funding Council as: “*benefits to one or more areas of the economy, society, culture, public policy and services, health, production, environment, international development or quality of life, whether locally, regionally, nationally or internationally*” [20]. Various studies have attempted to assess a range of impacts of research (especially health research) and/or attempted to identify facilitators and barriers of research use in policy-making. There are also a growing number of reviews of such studies [21–27]. While these are not explicitly studies of stakeholder engagement, many of them have identified some form of collaboration between researchers and users as one of the factors most likely to lead to the research making an impact. However, this wider range of literature does not go into detail in terms of analysing the nature of the processes of stakeholder engagement that leads to impact.

Studies specifically focusing on the impact of stakeholder engagement are less common, although it is a growing area of interest [28, 29]. Jolibert and Wesselink [2] found a few examples of impact, but suggested ways to increase impact through what they describe as sustained interactions. Concannon et al. concluded that approximately 20% of their study participants “*reported that stakeholder engagement improved the relevance of research, increased stakeholder trust... enhanced mutual learning by stakeholders, and researchers about each other, or improved research adoption*” ([13], p. 1697), whereas 6% reported improved transparency and 9% increased understanding of research processes. Also, while Forsythe et al. referred to a lack of evidence about impact, they also observed that “*Commonly reported contributions included changes to project methods, outcomes or goals; improvement of measurement tools; and interpretation of qualitative data*” ([30], p. 13). In the United States, the Center for Medical Technology Policy website makes a strong statement about the impact of stakeholder engagement: “*Including the perspectives of all key stakeholders has powerful benefits, enhancing both the short- and long-term relevance of clinical research efforts*” [31].

### Assessing the impact of stakeholder engagement: a new study

Given the diversity of stakeholder engagement and the thin evidence base for its impact, our study set out to identify a set of indicators that might be used to identify stakeholder engagement with potential for impact. We identified a study called the European study on Quantifying Utility of Investment in Protection from Tobacco (EQUIPT) and then conducted our own study, Stakeholder Engagement in EQUIPT (SEE-Impact) as a prospective study of stakeholder engagement running alongside. EQUIPT, a major European Commission (EC) – funded project, aimed to achieve impact through extensive stakeholder engagement. Both studies are briefly described in Box 1.

The results of the EQUIPT study have now been published [32, 33] and a full account of the main methods from SEE-Impact have been submitted for publication. Papers on the full findings are being finalised. Herein, our aim is to address the statement in our original funding proposal in 2013 that it should be possible to identify aspects of the stakeholder engagement (and perhaps other features of the processes) that might be viewed as intermediate indicators of the eventual impact achieved.

Our analysis of the complex and nuanced process of stakeholder engagement has resulted not in a list of indicators, but in a set of design principles. We hope that these design principles will help to inform the future development of stakeholder engagement as a mechanism for promoting research impact. These principles, rooted in both the existing literature and in the findings from our prospective study of stakeholder engagement, are intended to inform the planning and delivery of stakeholder engagement activities. It is anticipated that they will also provide a structure for building a narrative account of stakeholder engagement as part of an evaluation of an individual project or programme. They might also provide a starting point for the development of future indicators.

## Design principles for stakeholder engagement in research

The project team (comprising members of the SEE-Impact research team and collaborators from EQUIPT) met for a 2 day analysis workshop. One aim of the workshop was to begin to build a consensus among the team on what seemed to be the key design principles emerging from the SEE-Impact data and the on-going literature review. SEE-Impact data included observational data, interviews and document analysis. The research team continued to develop the principles through an ongoing period of deliberation, informed by the impact study and the literature. As part of this process, the principles were categorised into three groups, namely organisational, values and practices.

In this section, we first present empirical evidence from the SEE-Impact study that informed our development of the design principles. We then briefly summarise published evidence for each group of design principles in order to situate them in the wider literature.

### Design principles and empirical evidence from the stakeholder engagement in EQUIPT for impact (SEE-impact) study

The stakeholder engagement study (SEE-Impact) and the project being studied (EQUIPT) are described in Box 1. In terms of the organisational level principles, the EQUIPT project objectives for stakeholder engagement were clear, as set out in the proposal, protocol and project documents [34]. The key aims of stakeholder engagement activity were to access the knowledge and skills (described in the protocol as co-creation innovation in the working space) and to increase influence and impact (described in the protocol as dissemination innovation in the transfer space through stakeholder engagement).

In terms of values, the commitment to stakeholder engagement was more clearly demonstrated by some of the EQUIPT project team members than others. For some team members, previous successful experience of an interactive form of working with stakeholders had built a commitment to this particular way of working. It also provided experience of practical elements of working with stakeholders, but perhaps most importantly lived experience of the practical benefits of engagement. For other members of the team, too, working with stakeholders fitted closely with their ethos and values. For example, the Hungarian team talked about their pragmatic approach to research and the need to conduct useful and usable research, with stakeholder engagement being a key component. However, a small group within the wider project team did not seem committed to ensuring stakeholder engagement remained a core element of the project. They favoured a particular, individualised approach to stakeholders and, over time, partially reshaped the stakeholder engagement activities to something more akin to research participation (that is, taking part in a research study as a means of generating specific data as determined by researchers, rather than as co-producers of research). Finally, not all stakeholders identified by the project team were interested in engaging with the project. In particular, the lack of policy priority given to smoking cessation (the focus of the return on investment (ROI) tool) made engagement of policy stakeholders in the Netherlands very difficult to achieve.

In terms of practices, while the EQUIPT project protocol did set out how the stakeholder engagement would operate [34], there was not as much flexibility as the investigators would have liked in terms of the project plan and this had an impact on the nature of the stakeholder engagement activities. In particular, time intensive methods of engagement originally proposed in the protocol (particularly the large number of face-to-face meetings) began to look unrealistic to members of the team. The lack of flexibility came in part from the funder. The EC told the project team at an early point that there was no scope for negotiation around the project end date. Thus, initial delays in the project put a strain on the project timetable and deliverables. Members of the team proposed a shift from face-to-face meetings with stakeholders to Skype meetings in an effort to ‘catch up’. The technical team producing the new version of the ROI tool for roll out in Europe added to a sense of urgency in ‘speeding up’ the stakeholder engagement work with their need for data to feed into their work. Nevertheless, despite the practical difficulties, in EQUIPT, a significant amount of consideration had been given to stakeholder engagement, including planning how the input provided by stakeholders might be gathered, collated, analysed and used. Vokó et al. highlight that it is important to “*fully analyse several aspects of stakeholder engagement in research*” ([32], p. 15) and note that there is a tendency to ignore the value of early stakeholder engagement when it comes to development and transferability in the work of economic evaluation. EQUIPT’s careful consideration and the methods adopted facilitated a much more rigorous approach to stakeholder engagement than is often experienced.

### Design principles and supporting literature

The design principles for stakeholder engagement are organised into three groups, namely organisational, values and practices, albeit with some inevitable overlaps. We look at each category in turn, alongside a consideration of some of the relevant literature.

#### Organisational

1. Clarify the objectives of stakeholder engagement
2. Embed stakeholder engagement in a framework or model of research use
3. Identify the necessary resources for stakeholder engagement
4. Put in place plans for organisational learning and rewarding of effective stakeholder engagement
5. Recognise that some stakeholders have the potential to play a key role

##### Some examples from the literature

It is desirable to have a conceptual framework that situates stakeholder engagement as part of a plan for promoting research use in practice. Deverka et al. [6] proposed an ‘analytic-deliberative’ conceptual model for stakeholder engagement which “*illustrates the inputs, methods and outputs relevant to CER* [comparative effectiveness research]. *The model differentiates methods at each stage of the project; depicts the relationship between components; and identifies outcome measures for evaluation of the process*” ([6], p. 1). Furthermore, having a clear evaluation plan is considered critical. Concannon et al. recommended conducting “*evaluative research on the impact of stakeholder engagement on the relevance, transparency and adoption of research*” ([13], p. 1698). Esmail et al. argue that evaluations of stakeholder engagement should be “*designed a priori as an embedded component of the research process*” ([35], p. 142). They suggest that, where possible, evaluations should use predefined, validated tools. Jolibert and Wesselink [2] point out that linking stakeholders’ contributions with specific research objectives is important in order to establish when and how to engage and with whom. They argue that, at the recruitment stage, stakeholders should be made aware of, for example, their role/s, what they could contribute, costs in terms of time and effort, and benefits. Concannon et al. also conclude that funding is needed “*to account for the costs of implementing meaningful engagement activities*” ([7], p. 989).

In a Canadian study looking at stakeholder involvement in KT as a means of leading to more evidence-informed healthcare, Holmes et al. [36] identify a range of complexities which, they argue, need to be taken into account by funding schemes in order to meet funders’ and stakeholders’ expected ROI. Stakeholder involvement in research and implementing its findings is complex and time consuming, and the authors recommend an advocacy role where funders support a range of activities to address barriers to effective KT. These include carrying out an assessment of stakeholders’ KT needs “*to identify gaps and opportunities and avoid duplication of efforts*” ([36], p. 6). Kramer et al. [37] looked at the involvement of intermediary organisations as research partners on three interventions across four sectors, namely manufacturing, transportation, service and electrical utilities sectors. The authors describe the difficulties, benefits and challenges from the perspectives of both researchers and research partners and stress the importance of allowing the design of the protocol to be collaborative and flexible. Researchers need to honour, trust and respect their partners’ knowledge and expertise, and take into account their needs and priorities. Failure to meet these criteria will significantly dampen stakeholders’ enthusiasm. They also point out the importance of having a model of collaborative research with clear guidelines of how to conduct partnership research projects in order to further facilitate the use of research by practitioners. There would be an invested interest in “*the research question, design and findings, and this would prove to be very valuable as a knowledge transfer strategy*” ([37], p. 330).

The main literature on stakeholder analysis of policy-making is also useful for highlighting that some stakeholders have more potential to play a key role in the policy deliberations than others. For example, as part of their review of stakeholder analysis of health policy-making, Brugha and Varvasovszky [38] described an example in which the Hungarian Ministries of Finance and Industry were non-mobilised, high-influence, low-interest stakeholders in debates about public health interventions, but might, in some circumstances, become mobilised high-interest actors.

#### Values

6) Foster shared commitment to the values and objectives of stakeholder engagement in the project team
7) Share understanding that stakeholder engagement is often about more than individuals
8) Encourage individual stakeholders and their organisations to value engagement
9) Recognise potential tension between productivity and inclusion
10) Generate a shared commitment to sustained and continuous stakeholder engagement

##### Some examples from the literature

Concannon et al. [7] stress that researchers and stakeholders should be committed to the processes from the outset. Hinchcliff et al. [18] argue that it is important to define expectations and roles and provide time. Hering et al.’s [39] global study of water science and technology used stakeholder involvement in the objectives and approaches of the research for the co-production of knowledge as part of transdisciplinary research. Key aspects of particular value to the research included early identification and involvement of stakeholders, continuous engagement with stakeholders, and availability to stakeholders of supporting materials and in multiple languages. Mallery et al. recommend continuing to build trust with stakeholders “*throughout the engagement process*” ([5], p. 27).

#### Practices

11) Plan stakeholder engagement activity as part of the research programme of work
12) Build flexibility within the research process to accommodate engagement and the outcomes of engagement
13) Consider how input from stakeholders can be gathered systematically to meet objectives
14) Consider how input from stakeholders can be collated, analysed and used
15) Recognise identification and involvement of stakeholders is an iterative and ongoing process

##### Some examples from the literature

Forsythe et al. [30] highlight the importance of careful and strategic selection of stakeholders. As part of evidence and experience-based guidance to researchers and practice personnel about forming and carrying out effective research partnerships, Ovretveit et al. [40] developed a guide to categorise and describe types of partnerships or approaches to collaborative working. The guide sets out a framework for the roles and tasks, and the allocation of responsibilities for each partner involved. Roles and tasks are assigned to three main categories, namely questions, design and data, reporting and dissemination, and implementation and integration into organisation or policy. Concannon et al. [13] suggest the need to develop (and validate) stakeholder engagement tools to support engagement work. Forsythe et al. also stress the importance of “*establishing ‘parameters and expectations for roles’, giving stakeholders guidance, and allowing time for stakeholders to ‘get comfortable with their roles’ as important tasks*” ([30], p. 19).

The review of methods of stakeholder engagement conducted by Mallery et al. [5] identified a range of innovative methods and stressed the potential for engaging stakeholders at different points in the research process. The five methods highlighted for consideration were online collaborative forums, product development challenge contests, online communities, grassroots community organising and collaborative research. Jolibert and Wesselink [2] explored levels and types of stakeholder engagement in 38 EC-funded biodiversity research projects and the impacts of collaborative research on policy, society and science. They looked at how and when stakeholders were involved and the roles played, and argue that greater engagement throughout the whole of the research process, rather than, for example, at the dissemination stage, tends to lead to improved assessment of environmental change and effective policy proposals. Jolibert and Wesselink suggest, following Huberman’s [41] work in education, that it is desirable to have ‘sustained interactivity’ between researchers and users. Concannon et al. suggest that “*General principles can be drawn from community-based participatory research, which underscores that engagement is a relationship-building process*” ([7], p. 988). They found that, if bi-directional relationships are sustained over time, stakeholders can serve as ambassadors for high-integrity evidence even where the findings are contrary to generally accepted beliefs. Hinchcliff et al. point to the importance of “*building respect and trust through ongoing interaction*” ([18], p. 125). Forsythe et al. flag up the importance of continuous involvement and using in-person contact to build relationships [30]. They also stress the value in having a flexible approach that can adapt to the practical needs of stakeholders. A recent supplement of this journal edited by Paina et al. [4] also highlighted the importance of flexibility in making space for stakeholder engagement in research processes.

Based on the literature and the application of initial principles to our study, we have developed the elaborated design principles presented in Box 2.

## Conclusions

There is a growing interest in stakeholder engagement as a potentially promising approach to promoting research impact. There is also a developing literature mapping out who potential stakeholders might be (the ‘who’), considering approaches to stakeholder engagement (the ‘how’) and identifying rationales for stakeholder engagement (the ‘why’). In this paper, evidence from the literature around these dimensions has been combined with the findings from our study of stakeholder engagement in an EC-funded project to develop a set of design principles to inform future stakeholder engagement in research. The design principles encompass organisational factors, values and practices. We hope that the principles will be useful in planning stakeholder engagement activity within research programmes and in monitoring and evaluating stakeholder engagement. Active engagement of stakeholders may well shift our understanding of what research use looks like [39]. A next step will be to address the remaining gap in the stakeholder engagement literature concerned with how we assess the impact of stakeholder engagement on research use.

## Box 1: Studying stakeholder engagement in tobacco control policy

**EQUIPT: the European-study on Quantifying Utility of Investment in Protection from Tobacco**

The EQUIPT study set out to work with stakeholders to develop a tool to help government officials, policy-makers and healthcare providers across Europe examine the cost effectiveness and impact of anti-smoking initiatives. The tool was developed as part of a €2 million European Commission grant. An earlier version had already been piloted with local authorities around the United Kingdom, with users able to draw on specific circumstances, statistics and data to predict the impact of tobacco control in their particular regions. The successful stakeholder engagement in the United Kingdom work encouraged the research team to fully integrate stakeholder engagement into the European study. In this study, the following stakeholders were identified: National and European stakeholders consisting of policy-makers, academics, health authorities, insurance companies, advocacy groups, ministries of finance, national committees, clinicians and health technology assessment (HTA) professionals, and experts on smoking cessation and HTA. Ninety three stakeholders took part. They were engaged in a variety of ways, including through one-to-one interviews, Skype meetings and events. Much of the engagement activity focused on the development of the return of investment tool for application in different countries.

**SEE-Impact: Stakeholder Engagement in EQUIPT for Impact**

SEE-IMPACT was a 3-year prospective study awarded £157,000 from the United Kingdom’s Medical Research Council funding as part of their joint Methodology Programme with the National Institute for Health Research, earmarked to boost understanding of the impact of health-related studies on society and the economy. The study compared and contrasted the way the EQUIPT decision support tool was taken up in a further four European countries – Germany, Hungary, the Netherlands and Spain. The SEE-Impact study focused in particular on the ways in which stakeholders were engaged throughout the EQUIPT study. The study used a range of methods including interviews, surveys, observations and reviews of documents to develop a detailed understanding of how stakeholder engagement might work as a mechanism for promoting impact. An initial literature review on stakeholder engagement was used to distil a set of propositions for testing. Further details about the project can be found on the website of the MRC (now under United Kingdom Research and Innovation).

## Box 2 Design principles for stakeholder engagement

**Organisational**

**1) Clarify the objectives of stakeholder engagement**

The objectives might be one or more of accessing knowledge and skills; supporting interpretation of the results and drafting recommendations; supporting future influence and impact on policy and practice; increasing recruitment/enabling research; supporting transferability. The objectives need to be shared then among all parties.

**2) Embed stakeholder engagement in a framework or model of research use**

There are a number of models and frameworks designed to show how stakeholders might be engaged in a way that helps increase the chances of research being used in policy and practice, for example, the linkage and exchange model [9]

**3) Identify the necessary resources for stakeholder engagement**

Resources to consider are budget, time, skills and competences to manage engagement

**4) Put in place plans for organisational learning and rewarding of effective stakeholder engagement, for example, through appropriate evaluation of stakeholder engagement**

**5) Recognise that some stakeholders have the potential to play a key role**

Identify those stakeholders who are particularly interested in being engaged and those who are likely to be influential. Depending on the objective of stakeholder engagement, they may provide the most useful input, and are most likely to play a key role in using the results; their engagement should be especially encouraged

**Values**

**6) Foster shared commitment to the values and objectives of stakeholder engagement in the project team**

Ideally, make sure the commitment is there from the outset [6]

**7) Share understanding that stakeholder engagement is often about more than individuals**

Consideration needs to be given to stakeholders’ roles where they act as representatives – their power and influence within organisations and networks they represent and how these change over time

**8) Encourage individual stakeholders and their organisations to value engagement**

Support and build capacity for stakeholders and their organisations to engage

**9) Recognise potential tension between productivity and inclusion**

Engagement may lead to greater relevance and impact, but may have implications for productivity in meeting project objectives (for example, in a timely fashion). Engaging stakeholders, taking into account their needs and inputs and adjusting elements of the research project based on their feedback takes time and can slow down the research process

**10) Generate a shared commitment to sustained and continuous stakeholder engagement**

Project teams and stakeholders see the value of links between research producers and research users to build ongoing collaborations in order to meet the objectives

**Practices**

**11) Plan stakeholder engagement activity as part of the research programme of work**

This should be built into the project protocol or plan (see Pokhrel et al. [34])

**12) Build flexibility within the research process to accommodate engagement and the outcomes of engagement**

It will also be important to build in mechanisms to allow researchers to have the independence to articulate what is out of scope

**13) Consider how input from stakeholders can be gathered systematically to meet objectives**

The importance of some face-to-face contact and interactions should be considered

**14) Consider how input from stakeholders can be collated, analysed and used**

This important aspect of stakeholder engagement needs to be considered earlier than often happens

**15) Recognising identification and involvement of stakeholders is an iterative and ongoing process**

Ongoing interaction will be fostered by taking the time and creating the structures to build trustful relationships ([6, 12])

## Additional file

Additional file 1: SEE-Impact study literature search. (DOCX 17 kb)

## Abbreviations

EC: European Commission
EQUIPT: European-study on Quantifying Utility of Investment in Protection from Tobacco
KT: Knowledge translation
PPI: Patient and public involvement
ROI: Return on investment
SEE-Impact: Stakeholder Engagement in EQUIPT for Impact
