# Supplementary material for: How to engage stakeholders in research: design principles to support improvement
Source: Health Res Policy Syst. 2018 Jul 11;16:60. doi: 10.1186/s12961-018-0337-6 (PMC6042393; doi:10.1186/s12961-018-0337-6)
Supplement: Supplementary file 1 — SEE-Impact study literature search. (DOCX 17 kb) [file 12961_2018_337_MOESM1_ESM.docx]

**Additional file 1: SEE-Impact study literature search**

The literature search consisted of three main elements, the first two of which are described in more detail below. As the intention was to use the literature to inform the SEE-IMPACT study, rather than produce a stand-alone published review, we also continued to look for relevant papers during the course of the study. The three elements were:

1. A structured search of databases
2. A search of the websites of research funding bodies
3. Citation checking of key papers known to team members and snowballing

**Database search** 18/02/2014

This literature search aimed to find published papers on stakeholder engagement and research impact as part of a scoping exercise in the initial stages of the project. The search included four databases and Google Scholar. The search terms for each were as follows.

1. Pubmed
   Advanced search
   Search terms: “stakeholder engagement” AND “research impact”
2. Medline
   Search terms: “stakeholder engagement” OR “stakeholder involvement” OR “stakeholder participation” AND “research impact”
3. Web of Science
   Search terms: TS = ((“stakeholder engagement”) OR (“stakeholder involvement”) OR (“stakeholder participation”) AND (“research impact”))
   Scopus
   Title, Abstract, Keywords. All yrs

Search terms: “stakeholder engagement” OR “stakeholder involvement” OR “stakeholder participation” AND “research impact”

1. Google Scholar
   Search terms: “stakeholder engagement” OR “stakeholder involvement” OR “stakeholder participation” AND “research impact”

**Web search** (February 2014)

Two approaches were used: identifying relevant headings and snowballing; and searching the websites of research funders using the key term: stakeholder engagement. The main research funder sites searched were: Economic and Social Research Council, Medical Research Council, National Institute for Health Research, Wellcome Trust and Association of Medical Research Charities.
